# Unveiling synergistic QTLs associated with slow wilting in soybean (*Glycine max* [L.] Merr.)

**DOI:** 10.1007/s00122-024-04585-1

**Published:** 2024-03-19

**Authors:** Hakyung Kwon, Moon Young Kim, Xuefei Yang, Suk-Ha Lee

**Affiliations:** 1 Department of Agriculture, Forestry and Bioresources and Research Institute of Agriculture and Life Sciences, Seoul National University, Seoul, Republic of Korea; 2 Plant Genomics and Breeding Institute, Seoul National University, Seoul, Republic of Korea; 3 Key Laboratory of Herbage and Endemic Crop Biology of Ministry of Education, School of Life Sciences, Inner Mongolia University, Hohhot, 010030 China

## Abstract

**Key message:**

A stable *QTL qSW_Gm10* works with a novel locus, *qSW_Gm01*, in a synergistic manner for controlling slow-wilting traits at the early vegetative stage under drought stress in soybean.

**Abstract:**

Drought is one of the major environmental factors which limits soybean yield. Slow wilting is a promising trait that can enhance drought resilience in soybean without additional production costs. Recently, a Korean soybean cultivar SS2-2 was reported to exhibit slow wilting at the early vegetative stages. To find genetic loci responsible for slow wilting, in this study, quantitative trait loci (QTL) analysis was conducted using a recombinant inbred line (RIL) population derived from crossing between Taekwangkong (fast-wilting) and SS2-2 (slow-wilting). Wilting score and leaf moisture content were evaluated at the early vegetative stages for three years. Using the ICIM-MET module, a novel QTL on Chr01, *qSW_Gm01* was identified, together with a previously known QTL, *qSW_Gm10*. These two QTLs were found to work synergistically for slow wilting of the RILs under the water-restricted condition. Furthermore, the SNP markers from the SoySNP50K dataset, located within these QTLs, were associated with the wilting phenotype in 30 diverse soybean accessions. Two genes encoding protein kinase 1b and multidrug resistance-associated protein 4 were proposed as candidate genes for *qSW_Gm01* and *qSW_Gm10*, respectively, based on a comprehensive examination of sequence variation and gene expression differences in the parental lines under drought conditions. These genes may play a role in slow wilting by optimally regulating stomatal aperture. Our findings provide promising genetic resources for improving drought resilience in soybean and give valuable insights into the genetic mechanisms governing slow wilting.

**Supplementary Information:**

The online version contains supplementary material available at 10.1007/s00122-024-04585-1.

## Introduction

Drought stress is one of the significant problems hampering soybean yield, leading to a substantial reduction in the profitability of soybean cultivation up to 40% (Specht et al. 1999; Santini et al. 2022). Moreover, this issue is being exacerbated by the increasing severity and frequency of drought due to climate change. To mitigate drought damages, efforts have been made to increase the water availability of soybean plants under drought; irrigation farming directly supplies soybean water (Li and Troy 2018; Zhu et al. 2019; Luan et al. 2021) and soybean plants with high root to shoot ratios improve water accessibility or reduce water usage (Mwenye et al. 2018). However, the solutions are partial and incomplete because of an escalation in production costs and reduced pod set under normal conditions, respectively (Li et al. 2019). For genetic improvement of soybean varieties, therefore, it is necessary to identify and incorporate drought tolerance loci. It would allow soybeans to maintain yield potential and sustainability under normal conditions as well as in drought without adding additional costs to the farmer.

In the research for enhancing drought tolerance during recent decades, soybean genotypes that exhibit a slow-wilting phenotype are of considerable interest. Slow wilting is achieved by limiting the maximum transpiration rate under high vapor pressure deficit (VPD) conditions (Bunce 1984; Fletcher et al. 2007; Sinclair et al. 2017). By optimally regulating stomatal conductance in the balance of carbon dioxide intake with water conservation, slow-wilting genotypes are better able to retain soil moisture during drought conditions than fast-wilting ones (Fletcher et al. 2007; Sinclair et al. 2010). The phenotype allows them to delay reaching the permanent wilting point, which is a critical threshold at which plants lose turgor pressure and irreversibly wilt. A simulation model predicted that the trait potentially enhances soybean yields in drought conditions by over 75% across most regions of the USA without any yield loss even under water-sufficient conditions (Sinclair et al. 2010).

The first soybean genotype reported to possess a slow-wilting phenotype with limited transpiration under high VPD is PI 416937 (Fletcher et al. 2007). Its ability to limit transpiration was found to be related to a low hydraulic conductance between the leaf xylem and the guard cells (Sinclair et al. 2008), which possibly attributed to the insensitivity of aquaporin (also called a water channel) to silver (Sadok and Sinclair 2010a, b). Using a recombinant inbred line (RIL) population derived from crossing between PI 416937 (silver-insensitive) and Benning (silver-sensitive), quantitative trait locus (QTL) analyses detected seven loci associated with canopy wilting on chromosomes (Chrs) 02, 04, 05, 12, 14, 17 and 19 and four loci for limited leaf hydraulic conductance on Chrs03, 05, 10 and 12 (Carpentieri-Pipolo et al. 2012; Abdel-Haleem et al. 2012). Other soybean genotypes, such as Jackson (PI 548657) and KS4895 (PI 595081), have also been intensively investigated for the slow-wilting phenotype. Although both of them are considered to be drought-tolerant genotypes (https://www.soybase.org/projects/SoyBase.C2018.01.php) with no differences in canopy wilting (Charlson et al. 2009), significant differences in nitrogen fixation under drought stress have been observed (Hwang et al. 2015a). With transgressive segregation for canopy wilting in the RILs of KS4895 × Jackson, multiple loci were identified (Charlson et al. 2009). For transpiration responses to silver nitrate (aquaporin inhibitor), genetic loci were identified on Chr 10 and 12, using a RIL population of Jackson × KS4895 (Sarkar et al. 2022). QTLs associated with canopy temperature in the KS4895 × Jackson population and leaf temperature in the Jackson × KS4895 population were also reported (Bazzer and Purcell 2020; Sarkar et al. 2022). Under 15 different environments, QTL analyses for canopy wilting were additionally conducted using 5 different RIL populations including KS4895 × Jackson, KS4895 × Jackson, KS4895 × PI 424140, A5959 × PI 416937, and Benning × PI 416937 (Hwang et al. 2015b). Nine QTL clusters that concurred in at least two populations were identified on Chr 02, 05, 11, 14, 17 and 19. Except for a locus on Chr 14, which appears to be falsely positive, eight of the QTL clusters were refined through meta-analysis (Hwang et al. 2016). Recently, two landraces (PI 567690 and PI 567731) were found to exhibit a slow wilting trait by limiting maximum transpiration rate, while still possessing silver-sensitive aquaporins (Pathan et al. 2014). Novel QTLs responsible for the trait were identified using RIL populations derived from crosses of Pana × PI 567690 and Magellan × PI 567731 (Ye et al. 2020).

Despite such substantial efforts to identify genetic loci responsible for slow wilting and overall drought resilience, most studies have been conducted using a narrow genetic background primarily involving either PI 416937 and Benning or KS4895 and Jackson. Genetic factors or genes responsible for these traits remain unveiled. To gain a comprehensive understanding of this complex trait, it is necessary to include diverse slow-wilting genotypes with broader gene pools. In our previous study, where 11 soybean genotypes were screened under water-restricted conditions, a soybean genotype SS2-2 was identified for its distinct ability to conserve relative water content (RWC) in leaves, while the soybean elite cultivar Taekwangkong exhibited significantly lower RWC (Yang et al. 2023). SS2-2 is a unique biological material to enhance drought tolerance at the early vegetative growth stages, as spring drought is prevalent in Korea due to the monsoon climate. Therefore, this study is aimed to: (1) identify QTLs responsible for the slow wilting traits using the RILs derived from crossing between Taekwnag and SS2-2, (2) confirm the identified QTLs in other genetic backgrounds and (3) detect candidate genes in the QTLs for slow wilting.

## Materials and methods

### Plant materials

We used a RIL population developed from a cross between Taekwangkong and SS2-2 by single seed descent method to conduct QTL analysis for slow wilting, previously characterized for QTL analysis of resistance to Phomopsis seed decay (Sun et al. 2013). The maternal parent Taekwangkong is a Korean soybean elite cultivar (Kim et al. 1992), and the paternal parent SS2-2 is a supernodulating mutant of the wildtype Sinpaldalkong 2, generated by ethylmethane sulfonate mutagenesis (Lee and Lee 1998). In 2019, 110 RILs were subjected to drought stress induced by no watering. In 2020, however, additional 114 lines were advanced to F_9_ generations in the field of Seoul National University Farm, Suwon, Republic of Korea (N 37° 16′ 12.094'', E 126° 59′ 20.756''), resulting in producing a total of 224 RILs. In the next 2 years, 2021 and 2022, the 224 lines were phenotyped for wilting-related traits.

### Drought stress treatment by water restriction

The drought experiments of the Taekwangkong × SS2-2 population were carried out across a total of 11 independent environments, consisting of 4 trials in 2019, 3 trials in 2021 and 4 trials in 2022, from late April to July in the greenhouse at Seoul National University Farm. Four seeds of each line were initially sown in each pot, which contained 360 g of topsoil. The sterilized topsoil prevented soybeans from nodulation. Approximately 12 days after sowing, the seedlings were thinned to two healthy plants showing the same growth stage. The beginning of the drought stress treatment was in the V1 to V2 growth stages, approximately 17 days after sowing. Before the treatment, each pot was thoroughly watered three times at 15-min intervals to ensure complete soil saturation with water. The excess water was drained overnight, and the pots were subsequently covered with a plastic bag to prevent soil surface evaporation. Drought stress was imposed on the soybean plants by entirely withholding the water supply until phenotyping.

### Phenotypic evaluation

To verify if transpiration rate is implicated in the contrasting responses of the mapping parents to drought stress, we determined the fraction of transpirable soil water (FTSW) of Taekwangkong and SS2-2 under water restriction. The weight of each pot containing the parental lines was recorded as the initial pot weight, and the pots were weighed every 2 days after water restriction (DAWR) for up to 8 days. The final weight of the pot was measured after the soil in the pot was dried completely. FTSW was calculated based on the formula: (the weight of the pot on a specific day—the weight of the pot with totally dried soil)/(the initial weight of the pot—the final weight of the pot with totally dried soil). The decrease in the pot weight is considered to be attributed to the transpiration amount because evaporation on the soil surface was prevented (Devi et al. 2014).

With regard to wilting response to drought stress, wilting score and leaf moisture content were assessed in the Taekwangkong × SS2-2 population. These phenotypic traits were evaluated when approximately half of the RILs began exhibiting permanent tissue damage on at least one trifoliate leaf in a trial. The second trifoliate was selected for evaluation of wilting degree because it represented the plant's overall wilting level better than others. The degree of wilting was visually assessed using a 1 to 5 scale, where 1 = when leaves were in a vigorous status; 2 = when there was a loss of turgor in the second trifoliate; 3 = when leaves lost turgor, resulting in a shrunken and wrinkled leaf shape; 4 = when leaves showed partial permanent tissue damage; and 5 = when permanent tissue damage occurred throughout the entire second trifoliate (Fig. S1).

Measurement of leaf moisture content immediately followed grading wilting score. Second trifoliate leaves of two plants in a pot were picked and their petioles were removed. The weight of six leaflets was measured for the fresh weight (FW). These leaflets were then placed in a 60℃ oven for 72 h, after which the dry weight (DW) was recorded. Leaf moisture content was calculated using the formula, Leaf moisture content = (FW − DW) / FW.

### DNA extraction and bin map construction

Young healthy leaves of SS2-2 and their F_8_ RILs were collected for DNA extraction. The leaves were ground in liquid nitrogen and high-quality DNA was extracted using the GeneAll® Exgene™ Plant SV Kit (GeneAll Biotechnology, Seoul, Republic of Korea). Resequencing of SS2-2 was conducted with a paired-end approach using an Illumina HiSeq2500 instrument (Illumina Inc., San Diego, CA, USA) and the raw data were deposited on NCBI with the Sequence Read Archive (SRA) ID PRJNA1058168. For Taekwangkong, the resequencing data were obtained from NCBI under the SRA ID ERS3189953. For the RILs, genotyping-by-sequencing (GBS) was performed following the procedure described previously (Yoon et al. 2019; Lee et al. 2021).

Raw reads were initially processed with Trimmomatic v0.36 (Bolger et al. 2014) for quality control, and the barcode sequences of GBS reads were removed using the FASTX-Toolkit. The processed reads were mapped to the *G. max* reference genome sequence (Wm82.a2.v1) (Song et al. 2016) using the Burrows-Wheeler Aligner tool (Version 0.7.17) (Li 2013). SNPs were called from the mapped reads of the parental lines and the RILs using SAMtools and BCFtools (Li et al. 2009). Single nucleotide polymorphisms (SNPs) were filtered based on quality criteria, such as read depth greater than 2, a missing rate across the RILs less than 50% and mapping quality greater than 999 using VCFtools (Danecek et al. 2011). After that, SNPs that showed parental polymorphisms and were homozygous to each of the parental lines were selected with an in-house Python script. Missing or filtered SNPs were imputed using LinkImputeR (Money et al. 2017).

For the construction of a bin map with highly informative markers, recombination breakpoints were estimated using SNPbinner (Python 2.7) (Gonda et al. 2019), with a minimum bin size set to 30 kb. The marker distance of the genetic map was calculated using IciMapping 4.1 (Meng et al. 2015).

### QTL analysis

The phenotypic values of the wilting score and leaf moisture content were statistically analyzed with the R programming language (Team 2006). To account for and eliminate the environmental effect, mixed linear models were applied using the "minque" method (Wu 2019). For effective data visualization, the R packages "ggplot2" was used (Wickham et al. 2023).

QTL analysis was conducted by using IciMapping 4.1, with the inclusive composite interval mapping option with additive effects (ICIM-ADD). QTLs for the traits from a single year were detected using the BIP (additive, dominant and digenic epistasis genes in biparental populations) module with the averages of phenotypic values, after removing environmental effects. The interactions of QTLs with environments were estimated in the MET (multi-environment trial) module, using raw phenotype data from the parental lines and the RILs without prior data processing (Supplementary file 1). The mapping interval was established at 1 centimorgan (cM). The threshold for the logarithm of the odds (LOD) for each trait was determined by performing 1,000 permutation tests at a Type I error rate of 0.05. Loci containing marker intervals with an LOD score exceeding the determined threshold were identified as QTLs.

### Comparison with known drought-related QTLs

We sourced previously reported drought-related QTLs from SoyBase (https://soybase.org) (Brown et al. 2021) and obtained additional QTLs associated with transportation rate under VPD by literature survey (Sarkar et al. 2022), to compare with their genomic positions with those of QTLs identified in this study. Paralogous regions within the soybean genome were determined by using the MCScanX tool, with the generation of self-BLASTP output as the basis (Wang et al. 2012). The syntenic relationships between the paralogous regions of our QTLs and the overlaps with known drought-related QTLs were visualized utilizing a custom-made Python script.

### QTL validation in other soybean accessions

To validate the identified QTL in soybean genotypes with diverse genetic backgrounds, we obtained marker data of SoySNP50K from Soybase, for the 30 soybean accessions consisting of the 15 slowest and the 15 fastest-wilting ones among 373 soybeans from a previous GWAS study on drought resistance (Kaler et al. 2017). Using the genotypes of Taekwangkong and SS2-2 as pseudo-references, we converted the marker genotypes of these 30 accessions to either the Taekwangkong or SS2-2 type, then calculated the SNP index for each of the slow and fast wilting types. Any marker with a delta SNP index greater than 0.5 or less than − 0.5 represents genetic association with the wilting trait, in which positive and negative values indicate beneficial alleles from the SS2-2 type. The delta SNP index was calculated by subtracting the SNP index of the fast wilting type from that of the slow wilting type. For each SNP index, if any accessions followed the SS2-2 genotype, it was designated as + 1; if it followed the Taekwangkong genotype, it was designated as − 1. If the marker genotypes are not biallelic or identical between SS2-2 and Taekwangkong, it was designated as + 0. The resulting values were then divided by the number of accessions (in this case, 30).

### Survey of putative candidate genes

Sequence variation between Taekwangkong and SS2-2 in protein-coding genes inside the identified QTL regions was investigated using the reads obtained from resequencing. Additional filtering criteria for calling high-confident SNPs were applied: SNPs with a mapping quality greater than or equal to 30, a depth of coverage greater than or equal to 3, and homozygosity. SNP significance was determined using snpEff, which classifies variants into HIGH, MODERATE, LOW, and MODIFIER impact levels (Cingolani et al. 2012). We focused on SNPs with MODERATE and HIGH impacts, such as missense variations and stop gains potentially resulting in alterations of protein functions. Meanwhile, the expression patterns of the genes within the QTLs were compared between the mapping parents using transcriptome data from their leaves at the vegetative stage exposed to drought stress in the previous study (Yang et al. 2023). Differentially expressed genes (DEGs) were identified using criteria of a twofold expression change, logCPM cutoff value of 2 and *P* value < 0.05. Genes with functional nucleotide variants or showing differential expression were considered as putative candidate genes for a corresponding QTL if their Arabidopsis orthologs had been previously reported to be involved in drought stress. For Glyma.01G098400 in the identified QTL *qSW_Gm01*, its putative promoter region was estimated by aligning the 1 Mb upstream sequence of its transcription start site (TSS) with the known promoter sequence of the Arabidopsis orthologs using ClustalW (Thompson et al. 1994). To identify paralogs of Glyma.10G019000 in the identified QTL *qSW_Gm10*, all genes encoding a member of the ATP-binding cassette C (ABCC) subfamily in the soybean and Arabidopsis genomes were subjected to MEGA5 program to draw a phylogenetic tree (Tamura et al. 2011). The soybean genes in a minimum clade harboring the nearest Arabidopsis ortholog are considered paralogs of the Glyma.10G019000. The structures of genes were visualized using TBtools (Chen et al. 2020). The domains in the Glyma.10G019000 are predicted using SMART (Schultz et al. 2000).

## Results

### Evaluation of wilting responses to restricted water supply in Taekwangkong, SS2-2, and their RILs

**Fig. 1:**
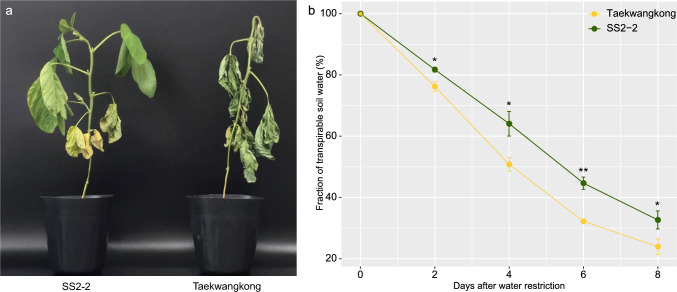
Comparative response to drought stress in the parental lines, Taekwangkong and SS2-2, at the early vegetative stage. **a** Phenotypic appearances of SS2-2 and Taekwangkong at 10 days after water restriction (DAWR). SS2-2 and Taekwangkong showed slow and fast wilting, respectively. **b** Reduction of a fraction of transpirable soil water (FTSW) in the parental lines according to DAWR. Green and yellow lines indicate slow wilting SS2-2 and fast wilting Taekwangkong, respectively. * and ** denote significance at the *P* value of one-tailed Student’s *t* test < 0.05 and 0.01, respectively

The mapping parents, Taekwangkong and SS2-2 showed contrasting responses of their aerial parts to 10-day drought stress, fast wilting and slow wilting, respectively (Fig. 1a), coincident with the results in our previous study (Yang et al. 2023). At 10 DAWR, the SS2-2 leaves in the lower positions only lost turgidity, in contrast, the Taekwangkong leaves dried to such an extent that the tissues experienced permanent damage, potentially irreversible even by rehydration. The imposition of drought stress reduced FTSW continuously in the two cultivars (Fig. 1b), but SS2-2 showed higher FTSW than Taekwangkong for 10 DAWR and their differences widened toward the end of the stress treatment (Fig. 1b). From 2 to 8 DAWR, FTSW ranged from 81 to 33% in SS2-2 and from 76 to 24% in Taekwangkong. These results indicate that SS2-2 shows slower water use attributed to reduced transpiration than Taekwangkong after water was withheld.

**Fig. 2:**
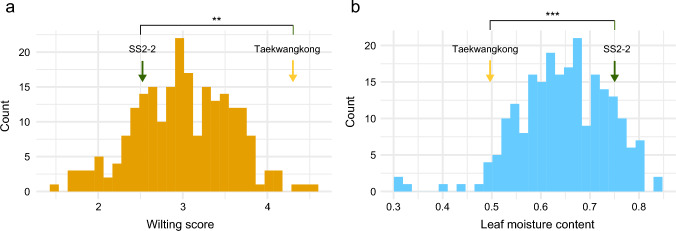
Phenotypic distribution of wilting score (**a**) and leaf moisture content (**b**) in the Taekwangkong x SS2-2 population. Green and yellow arrows indicate drought-tolerant SS2-2 and drought-sensitive Taekwangkong, respectively. *, ** and *** denote significance at *P* value of one-tailed Student’s *t* test < 0.05, 0.01, and 0.001, respectively

We observed significant differences between the mapping parents in terms of wilting score and leaf moisture content as traits related to drought tolerance (Fig. 2). Taekwangkong had a wilting score of 4.30, significantly higher than 2.52 of SS2-2, with the *P* value of the *t*-test less than 0.001. In the case of leaf moisture content, SS2-2 significantly exceeded Taekwangkong (0.50) with a value of 0.75 (*P* = 0.005). The phenotypic data for both of the traits showed continuous distribution of quantitative traits in the RIL population of Taekwangkong × SS2-2 (Fig. 2), and dozens of RILs had phenotypic values exceeding those of the parents (Fig. 2), meaning that transgressive segregation occurred for the two traits toward both maternal and paternal directions. The 38 lines displayed slower wilting than SS2-2, with a lower wilting score and higher leaf moisture contents. The population presented a mean wilting score of 2.30 ± 0.57 (standard deviation) under the drought stress treatment, with a range of 1.46 to 4.52. For leaf moisture content, the population showed a wide range of phenotypic variations (0.30 to 0.83) with a mean of 0.65 ± 0.09. Broad sense heritability estimates were moderate for both of the wilting score (0.52) and moisture content (0.47). A strong negative correlation was observed between the two traits, as evidenced by a Pearson correlation coefficient of − 0.71. There were weak but significant correlations between the annual phenotypic data of each trait, except for a relationship between moisture contents observed in 2019 and 2022 (Fig. S2, Table S1).

**Table 1:** QTLs for wilting score and leaf moisture content in the RIL population derived from Taekwangkong × SS2-2 with ICID-MET module

Trait	Locus	LOD threshold	Chr	Position (cM)	Flanking markers	LOD (A^a^ + AbyE^b^)	PVE^c^ (A + AbyE)	Add.^d^	Source of beneficial allele	No. of genes
Wilting score	*qSW_Gm10*	8.25	10	6	Bin_10_1686946, Bin_10_2097501	18.36 (11.11 + 7.25)	11.26 (5.05 + 6.21)	0.16	SS2-2	100
Leaf moisture content	*qSW_Gm01*	7.51	1	36	Bin_1_33110420, Bin_1_34541239	7.79 (4.42 + 3.36)	10.30 (2.90 + 7.39)	− 0.02	SS2-2	70
	*qSW_Gm10*	7.51	10	6	Bin_10_1686946, Bin_10_2097501	15.45 (6.49 + 8.96)	9.10 (4.22 + 4.88)	− 0.02	SS2-2	100

^a^Additive and dominance effects

^b^Additive and dominance by environment effects

^c^PVE the percentage of phenotypic variance explained by the QTL

^d^Add the allelic additive effect

### Bin map construction

To construct a bin map of the Taekwangkong × SS2-2 population, we generated a total of 16 and 17 Gb sequence data in Taekwangkong and SS2-2, respectively, achieving an average depth of 14.6× and 13.5× and coverage of 91.2 and 92.3%, respectively (Table S2). We identified 1,406,133 SNPs between the parental lines. Among 3,100,364 SNPs detected in the RILs from the GBS data, we selected 10,983 high-quality SNPs (Table S3). By detecting recombination breakpoints and using every bin as a marker, a total of 1383 bins were integrated into a bin map spanning 2043 cM across the soybean genome (Table S4). The markers were evenly distributed across 20 chromosomes. The chromosome length ranged from 71.4 to 135.5 cM, with an average marker distance of 1.48 cM.

### QTL identification for slow wilting

A total of ten QTLs were identified for the wilting-related traits in the three years by the BIP module, distributed across eight chromosomes (Chr01, 03, 04, 05, 06, 07, 09, 10) (Table S5). Despite the significant correlation between wilting score and leaf moisture content, 8 loci out of them were detected only once in a single year for a single trait, with beneficial alleles from either of the parents. Three QTLs, *qSW_Gm03*, *qSW_Gm04_1* and *qSW_Gm04_2,* were associated with wilting scores in 2019. Interestingly, *qSW_Gm04_1* and *qSW_Gm04_2* on Chr4 are only 3 cM apart, with high LOD values of 28.1 and 19.1, but their beneficial alleles come from different parental lines, respectively. In 2021, *qSW_Gm05* and *qSW_Gm06_1* were identified for the wilting score, and *qSW_Gm06_2* and *qSW_Gm09* for leaf moisture content. Beneficial alleles at *qSW_Gm06_1* and *qSW_Gm06_2*, which are 6 cM apart from each other, come from Taekwangkong, probably counted as the same locus. At *qSW_Gm07* in 2022, Taekwangkong also offered a positive allele for leaf moisture content.

**Fig. 3:**
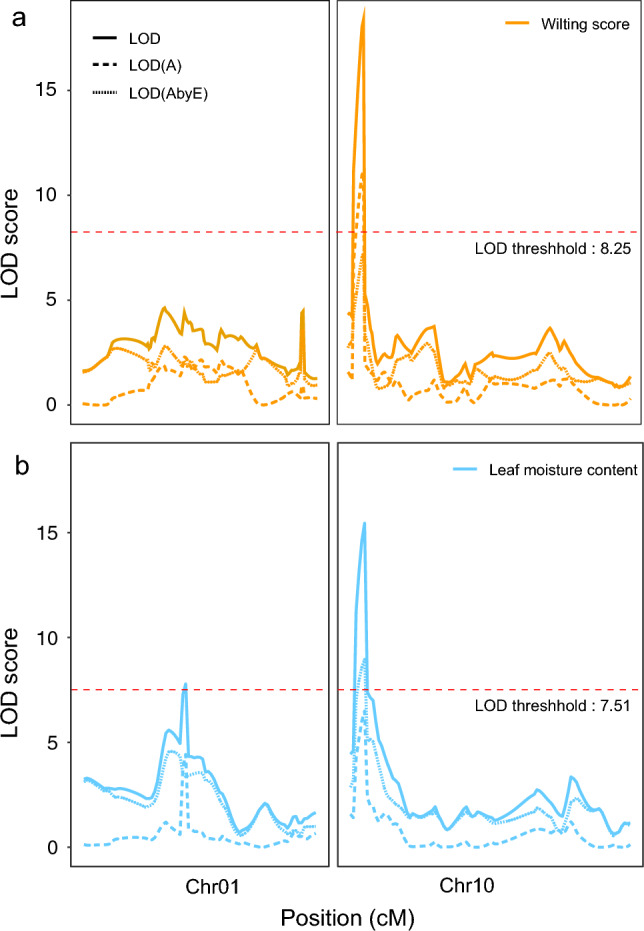
Distribution of LOD scores of QTLs associated with wilting score (**a**) and leaf moisture content (**b**). Solid, dashed and dotted lines represent the LOD scores for all effects, additive and dominance effects (A) and additive and dominance effects by environment effect (AbyE)

The remaining two loci *qSW_Gm01* and *qSW_Gm10* on Chr 1 and Chr 10 were distinct as they were associated with both of the traits in a single year or with multiple years for a single trait, respectively. These two loci were also revealed to be stable loci controlling slow wilting by QTL analysis under the MET module (Table 1, Fig. 3). The *qSW_Gm01* locus for leaf moisture content showed a LOD score of 7.8, which explained 10.3% of phenotypic variation (Table 1). The LOD scores of *qSW_Gm10* were 18.4 and 15.5 for wilting score and leaf moisture content, respectively; it explained 11.3 and 9.1% of phenotypic variations for the two traits, respectively (Table 1). Additive effects showed beneficial alleles to delay wilting at the two loci were contributed from only SS2-2. The high LOD scores of *qSW_Gm01* and *qSW_Gm10* suggest that they appear to play a role as core factors across multiple environments. Meanwhile, the values of phenotypic variation explained by additive and dominance by environment effect, PVE (AbyE), were larger than those by additive and dominance effects, PVE(A) for corresponding QTLs (Table 1), suggesting considerable interaction of the loci with environments for the control of slow wilting.

### Comparison with the reported wilting-related QTLs

**Fig. 4:**
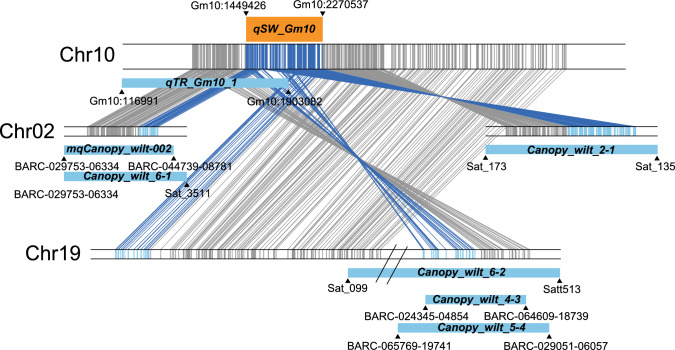
Comparison of *qSW_Gm10* with other known drought-related QTLs. Vertical bars on chromosomal segments represent protein-coding genes, linked with paralogous genes within syntenic regions. Genes in the *qSW_Gm10_*region and their paralogous genes are represented by blue and light blue colors, respectively. Orange and blue rectangles represent QTL intervals; black triangles indicate markers flanking the QTLs

We compared our identified QTLs with previously reported wilting-related loci to know if they were novel or not. None of the wilting-related, or drought-related QTLs overlap with the *qSW_Gm01*, and its syntenic regions do not harbor any known QTLs, indicating the discovery of a novel loci. On the other hand, the genomic region of *qSW_Gm10* partially overlaps the reported QTL (*qTR_Gm10_1*) associated with limited transpiration rate and sensitivity to an aquaporin inhibitor, silver nitrate (AgNO_3_) (Fig. 4) (Sarkar et al. 2022). Moreover, three genomic regions paralogous to *qSW_Gm10* also contain several previously reported QTLs for canopy wilting: *Canopy_wilt 2–1* on Chr02 (Abdel-Haleem et al. 2012), *Canopy_wilt 6–1* on Chr02 (Hwang et al. 2015b), *Canopy_wilt 4–3*, *Canopy_wilt 5–4* and *Canopy_wilt 6–2* on Chr19 (Hwang et al. 2015b).

### QTL validation using different germplasms

**Fig. 5:**
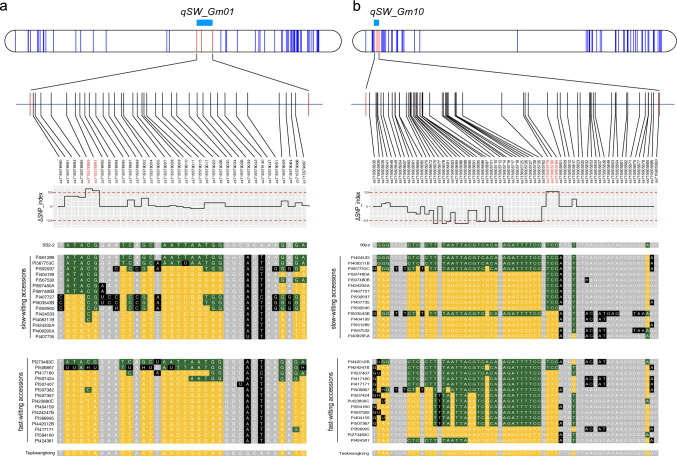
SNP index and haplotypes in the identified QTL regions for *qSW_Gm01* (**a**) and *qSW_Gm10* (**b**) across the 15 slowest and fastest wilting soybean genotypes with different genetic backgrounds using SoySNP50K marker data. In the chromosomes represented by rounded rectangles, vertical blue lines indicate SNPs used in the bin map construction of the Taekwangkong × SS2-2 RIL population. Red lines denote SNPs marking the QTL regions. Below each chromosome, an enlarged view of the QTL region is presented, overlaid with vertical black line for SNPs derived from the SoySNP50K dataset. Marker names are listed below these lines, followed by the Δ SNP index, which illustrates the bias in SNP genotypes according to the wilting types. The SNP alleles of Taekwangkong, SS2-2, and slow- and fast-wilting accessions are represented by white letters. Green and yellow blocks indicate nucleotides matching the slow-wilting SS2-2 and fast-wilting Taekwangkong, respectively. Grey blocks show no nucleotide difference between the parents, and black blocks exhibit nucleotides in 30 diverse soybean accessions different from those of both parents (Colour figure online)

To validate the two major QTLs for slow wilting across diverse genetic backgrounds, we investigated allelic association at the loci between the 15 lowest and highest accessions for canopy wilting scores reported in a previous study (Kaler et al. 2017) (Fig. 5). In the *qSW_Gm01* region, the markers ss715578989 and ss715578991 (from SoySNP50K) showed allelic segregation, with a delta SNP index greater than 0.5, probably considered as a true positive supported by the existence of expanded allelic segregation to the upstream of *qSW_Gm01* (data not shown). In the *qSW_Gm10* region, three markers ss715605778, ss715605780, and ss715605784 displayed a genetic association with the wilting type with a delta SNP index greater than 0.5. Ten out of the 15 slow-wilting accessions displayed the same genotype as SS2-2, and 13 out of the 15 fast-wilting accessions exhibited the Taekwangkong genotype. Moreover, 19 out of the rest informative 33 markers exhibit genetic association, albeit in the opposite direction. This result suggests that *qSW_Gm01* and *qSW_Gm10* may operate stably across different genetic backgrounds.

### Effects of allelic combinations from *qSW_Gm01* and *qSW_Gm10*

**Fig. 6:**
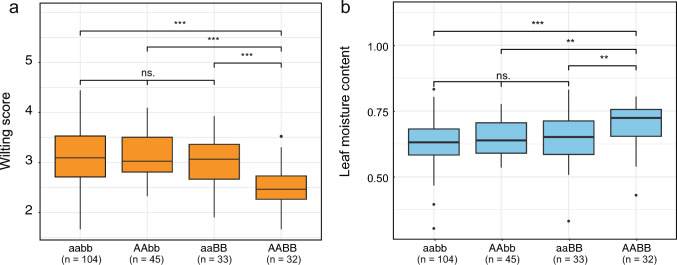
The effects of the combined alleles at two loci, *qSW_Gm01* and *qSW_Gm10*, on the wilting score and leaf moisture content in the RIL population. Beneficial alleles from SS2-2 at *qSW_Gm01*and *qSW_Gm10* are represented by "A" and "B", respectively, while deleterious alleles from Taekwangkong at the two loci are denoted by “a” and “b”, respectively. ** and *** indicate significant differences between the allele combinations for *P* < 0.01 and 0.001 by using the Wilcoxon test, respectively. *ns* not significant

To estimate the effect of combined alleles from the two major QTLs *qSW_Gm01* and *qSW_Gm10*, we characterized genotypes of the RILs for the loci. The genotypes having beneficial alleles derived from SS2-2 at *qSW_Gm01* and *qSW_Gm10* were designated as ‘‘AA” and ‘‘BB”, and those from Taekwangkong were designated as ‘‘aa” and ‘‘bb”, respectively. RIL lines were classified into four groups according to the genotypes: 104 lines for aabb, 45 for AAbb, 33 for aaBB, and 32 for AABB (Fig. 6). Multiple comparison tests showed that the mean wilting score (2.5) of RILs with genotype ‘‘AABB” was significantly lower than those of lines with genotype “AAbb” (3.1) and “aaBB” (3.0) as well as ‘‘aabb” (3.1) (Fig. 6). Lines with genotypes “AAbb” and “aaBB”, with a single beneficial allele at either *qSW_Gm01* or *qSW_Gm10* did not show significantly lower wilting scores than that of genotype “aabb”. Similarly, the mean leaf moisture content (0.70) of RILs with genotype “AABB” showed a significant difference from those of three genotypes “aaBB” (0.64), “AAbb” (0.65) and “aabb” (0.63), but there was no significant difference among these three genotypes (Fig. 6). Statistical analyses in the individual years showed similar results that only RILs with genotype “AABB” displayed significantly different phenotypic values for slow wilting compared to the lines with the other genotypes in 2021 and 2022 (Fig. S3); in 2019, leaf moisture content of RILs with genotype “AABB” is significantly higher than that of “aabb” and “AAbb” by multiple comparison test, but there was no significant difference by Duncan test. Notably, these results showed the coexistence of both beneficial alleles elevated phenotypic values of the wilting score and moisture content greater than the sum of additive effects of each allele, indicating that *qSW_Gm01* and *qSW_Gm10* may interact with each other with a synergistic effect.

### Survey of candidate genes

To identify candidate genes within the discovered major QTLs, we employed two strategies. The first strategy focused on DNA mutations that could potentially impair protein function. As candidate genes, we considered ones with significant variants in the coding sequence (CDS) that are functionally expressed, especially those whose Arabidopsis orthologs have been reported to function related to drought resistance. The second strategy involved variants affecting the expression levels of drought responsive genes. We first selected genes that showed significant expression differences between the parental lines under drought conditions, but not under control conditions. Among them, genes with variants in their cis-elements were further selected and their Arabidopsis orthologs were considered if involved in drought stress.

#### Survey of candidate gene for *qSW_Gm01*

The QTL *qSW_Gm01* (between Bin_1_33110420 and Bin_1_34541239) harbors three SNPs (Gm01:32,691,301, Gm01:33,529,540 and Gm01:35,552,939) which serve as recombination breakpoints among the RILs. From their physical positions spanning 2.86 Mb, 70 genes were located in the *qSW_Gm01*, 38 of which have parental nucleotide polymorphisms in the genic region (Fig. S4, Table S6). Among 19 genes embracing SNPs with significant impacts, only 2 genes (Glyma.01G099600 and Glyma.01G104800) were found to be functionally transcribed from our previous RNA-seq data (Table S7) (Yang et al. 2023); Glyma.01G099600.1 and Glyma.01G014800.1 encode autophagy-related protein 1c (ATG1C) and galactose oxidase-like2 (GOXL2), respectively. However, there is no report about the functions of their Arabidopsis orthologues related to drought stress.

No DEG among genes inside the QTLs *qSW_Gm01* was detected between the mapping parents under the control (well-watered) condition. In *qSW_Gm01*, five genes (Glyma.01G098400, Glyma.01G098700, Glyma.01G099800, Glyma.01G100200 and Glyma.01G104100) showed differential expression between Taekwangkong and SS2-2 under drought condition (Table S7), of which Glyma.01G099800 and Glyma.01G100200 had no SNP detected in 2 kb upstream or downstream. Only in the case of Glyma.01G098400, which is upregulated in SS2-2, the Arabidopsis ortholog AT2G28930 (Arabidopsis protein kinase 1b, APK1b) was reported to be related to drought stress with its function in light-induced full stomata opening (Elhaddad et al. 2014). However, the variants in the putative promoter region of Glyma.01G098400 could not be investigated, because the region with low GC content (< 25%) and repetitive sequences was poorly sequenced in our NGS data, especially in Taekwangkong; there is the 449 bp region of zero coverage in Taekwangkong, while SS2-2 has no zero coverage region and 373 bp of low depth (≤ 3) region. Based on these findings, Glyma.01G098400 (PK1b) has been selected as a candidate gene for *qSW_Gm01*.

#### Survey of candidate gene for *qSW_Gm10*

**Fig. 7:**
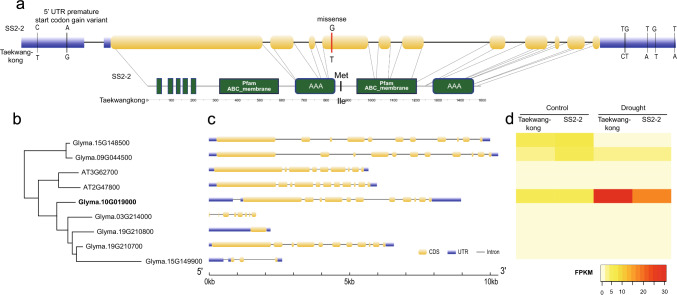
Identification of Glyma.10G019000 as a putative candidate gene for *qSW_Gm10*. **a** DNA sequence variants (upper) and amino acid change (lower) of Glyma.10G019000. In the gene structure (the upper diagram), yellow rounded rectangle, blue rectangle and black line represent CDS, UTR and intron, respectively. For the protein structure (the lower diagram), five transmembrane regions, two ABC_membrane domains and two ATPases associated with a variety of cellular activities (AAA) domains are detected from the TMHMM v2.0 program, Pfam and SMART database, respectively. **b** Phylogenetic tree of homologs of Glyma.10G019000 in Arabidopsis and soybean. **c** Gene structure of Glyma.10G019000 and its homologs **d** Expression level of Glyma.10G019000 and its paralogs under control and drought condition. The expression level of Arabidopsis orthologs of Glyma.10G019000 is not shown

The QTL *qSW_Gm10* in the interval of Bin_10_1686946 and Bin_10_2097501 corresponds to the 821 kb genomic regions from three SNPs, Gm10:1,449,426, Gm10:1,924,466, and Gm10:2,270,537, containing 100 genes. Among 45 polymorphic genes between the parental lines, 14 genes had SNPs with significant impacts (Fig. S4, Table S6). Five genes (Glyma.10G017700, Glyma.10G017900, Glyma.10G019000, Glyma.10G019300, and Glyma.10G021800), functionally expressed, showed nonsynonymous missense mutations in exons between the parental lines (Table S6): 3 SNPs in Glyma.10G017700 (poly(ADP-ribose) polymerase 2, PARP2), 2 in Glyma.10G021800.1 (degradation of periplasmic proteins 2, DEG2), 1 in each of Glyma.10G017900 (photolyase/blue-light receptor 2, PHR2), Glyma.10G019000 (multidrug resistance-associated protein 4, MRP4) and Glyma.10G019300 (AAA-type ATPase family protein). For only Glyma.10G019000, its Arabidopsis ortholog AT2G27800 has been reported for its function in drought stress (Klein et al. 2004; Gong et al. 2022). Compared to SS2-2, we detected multiple variants in the genic region of Glyma.10G019000 in Taekwangkong, including a missense variant in which the amino acid at position 87 changed from Met to Ile and a premature start codon gain variant in 5’ untranslated region (UTR) region (Fig. 7).

*qSW_Gm10* contained seven DEGs (Glyma.10G018300, Glyma.10G018700, Glyma.10G021300, Glyma.10G021700, Glyma.10G022900, Glyma.10G023200, and Glyma.10G024200) between the mapping parents under drought stress (Table S7), with none of which differentially expressed between the mapping parents under the control (well-watered) condition. However, no orthologs of these genes have been found to function in drought tolerance. Based on these findings, Glyma.10G019000 (MRP4) has been selected as a candidate gene for *qSW_Gm10*.

## Discussion

The typical soybean sowing season in Korea is from late May to mid-June, in which soybean is intercropped with barley or double-cropped soybean is planted as the second crop after barley is harvested. During the spring, however, Korea suffers from widespread and severe drought almost every year like other East Asia counties, caused by two main causes: monsoonal circulation resulting in large seasonal variations in precipitation and northerly wind along its east coastline (Zhang and Zhou 2015). Since precipitation in spring is only 19% (237.3 mm) of annual precipitation, a shortage of water in this period greatly harms soybean growth in the early vegetative growth stages until the summer monsoon rains begin (Kim et al. 2018). The onset of the summer monsoon leads to summer rainfall (638.7 mm) accounting for 50% of the annual precipitation (1237.4 mm) in Korea (Kim et al. 2018). Major limiting factors for soybean yield in Korea were reported to be shortages of soil moisture in June as well as September (Shin et al. 2015). Related to global warming, furthermore, the occurrence and durations of spring drought in East Asia including Korea show a tendency to increase with increased temperature, abnormal precipitation and delayed monsoon (Jumrani and Bhatia 2018; Kim et al. 2018; Leng and Hall 2019; Chun et al. 2021). The average annual temperature has risen by 0.18 °C per decade, with an increase of 0.24 °C each spring compared to 0.08 °C in summer. Moreover, the peak of the first precipitation in the recent 30 years (1988–2017) in Korea was delayed from July 8 to July 14 in the last 30 years (1912–1941) (Kim et al. 2018). According to the preliminary results on the exploration of soybean yield changes in Korea due to extreme climate anomalies, a 5–10% and 20–22% decrease in cumulative precipitation in June would reduce soybean yield by 10–14% and 47–49%, respectively (Chung et al. 2015). Therefore, the slow wilting characteristic of the Korean soybean cultivar SS2-2 under drought stress at the early vegetative stages is of great importance for preventing yield loss through the development of drought-tolerant varieties, with further saving productive costs.

The QTL *qSW_Gm01* on Chr1 was identified as a novel QTL for slow wilting (Table 1 and Fig. 3), not overlapping with any other previously reported loci. Using the SoySNP50K data, we investigated nucleotide variants within *qSW_Gm01* in the mapping parents used in the previous studies on slow wilting: Jackson and KS4895 (Hwang et al. 2015b, 2016; Bazzer and Purcell 2020; Sarkar et al. 2022), and Benning and PI 416937 (Carpentieri-Pipolo et al. 2012; Abdel-Haleem et al. 2012; Hwang et al. 2015b, 2016). There was a lack of variants on the locus between Jackson and KS4895. SNP markers with different alleles for *qSW_Gm01* were detected between Benning and PI 416937 (data not shown), but the genetic map of the population of these genotypes consisted of only 276 SSR markers, probably having low resolution to identify *qSW_Gm01*. In this study, we used SS2-2 as a paternal line, which is a unique resource to contain favorable alleles such as supernodulation and resistance to bacterial leaf pustule (Lee and Lee 1998; Kim et al. 2012), and constructed the high-resolution bin map (Table S4), leading to successful identification of the novel locus responsible for slow wilting on Chr01. *qSW_Gm01* was additionally validated in 15 soybean accessions that ranked as the highest and the lowest genotype for canopy wilting in a previous study, respectively (Fig. 5).

The QTL *qSW_Gm10* on Chr10 is co-localized with the previously reported QTL *qTR_Gm10_1* (Fig. 4). In the RIL population of Jackson x KS4895, *qTR_Gm10_1* was identified for transpiration rate under the silver nitrate aquaporin inhibitor treatment (TRAgNO_3_) and decrease in transpiration rate by silver nitrate (TRH_2_O—TRAgNO_3_), but it was also associated with transpiration rate under H_2_O condition (TRH_2_O) (Sarkar et al. 2022). Moreover, the LOD score and PVE value of TRH_2_O—TRAgNO_3_ were lower than those of TRH_2_O and TRAgNO_3_ (Sarkar et al. 2022). In another two RIL populations derived from new slow-wilting plant introductions (PIs), Pana × PI 567690 and Magellan × PI 567731, four and two QTLs were identified for slow wilting, respectively (Ye et al. 2020). A QTL from PI 567731 was positioned in the interval of Gm10_43894668/Gm10_44744804 on Chr10 (Ye et al. 2020), but being far away from our QTL *qSW_Gm10* (Gm10:1,449,426. Gm10:2,270,537). PI 567690 and PI 567731 exhibited sensitivity in transpiration to the AgNO_3_ treatment, unlike silver-insensitive PI 416937. Meanwhile, SS2-2 showed the same alleles at three adjoining maker positions (of SNP50Kchip) in *qSW_Gm10* as Jackson rather than KS4895, from which alleles for the *qTR_Gm10_1* QTL were associated with insensitivity in transpiration to silver nitrate relative to Jackson (Sarkar et al. 2022). Considering these previous reports, it is unclear if a positive allele from SS2-2 at *qSW_Gm10* for slow wilting is relevant to the lack of silver-sensitive aquaporins. For a deep understanding of slow wilting, therefore, it is necessary to examine how limited transpiration rate is related to aquaporin regardless of sensitivity to silver or if other mechanisms are involved in slow wilting under drought stress (Ye et al. 2020).

Beneficial alleles from SS2-2 at the two QTLs *qSW_Gm01* and *qSW_Gm10* had a synergistic effect on wilting score and moisture content, exceeding the sum of effects from individual alleles, but individual alleles had no significant additive effect on the traits (Table 1, Fig. 4). Although *qSW_Gm01* or both of them were not detected as main QTLs by BIP analysis, interestingly, synergism of combined beneficial alleles (AABB) was observed in all individual years, (Table S5, Fig. S3). In 2022, when *qSW_Gm01* was not detected as a main QTL, RILs with SS2-2 allele only at *qSW_Gm10* (aaBB) showed little difference in wilting score and moisture content compared to RILs without it (AAbb and aabb) but RILs of “AABB” showed a significant difference from RILs with the remaining genotypes (aaBB, AAbb and aabb) (Fig. S3). In 2019, when both *qSW_Gm01* and *qSW_Gm10* were not main QTLs (Table S5), the SS2-2 allele (“AA”) at *qSW_Gm01* showed significant additivity in wilting score in the case of “BB” at *qSW_Gm10*. Likewise, the SS2-2 allele (“BB”) at *qSW_Gm10* significantly enhanced moisture content in the presence of the *qSW_Gm01* allele of SS2-2 (“AA”) (Fig. S3). Regarding *qSW_Gm10* is consistently identified as a main QTL with high LOD, it can be inferred that *qSW_Gm10* plays a pivotal role in creating a synergistic effect on slow wilting with *qSW_Gm01*.

Glyma.01G098400 and Glyma.10G019000 are highly likely to be the candidate genes for *qSW_Gm01* and *qSW_Gm10*, respectively. Glyma.01G098400 encoding protein kinase 1B (PK1B) is upregulated in SS2-2 by drought stress with no responses in Taekwangkong. A knockout mutant of its Arabidopsis orthologs, AT2G2893, exhibited reduced stomatal aperture under light conditions, causing decreased transpiration and increased relative water content under drought conditions (Elhaddad et al. 2014). Glyma.10G019000 encodes a multiple resistance protein 4 (MRP4), also named ATP-binding cassette C4 (ABCC4) protein, upregulated in both parental lines by drought stress (Fig. 7). Malfunction of the protein in Taekwangkong, however, might be caused by a missense mutation from Met to Ile at positions 873 as well as multiple 5’ and 3’ UTR variants, compared to SS2-2 (Fig. 7a). In addition, we found 6 paralogs of Glyma.10G019000 in the soybean genome (Fig. 7b, c), which were little expressed in leaves both under control and drought conditions (Fig. 7d), consistent with a previous study (Mishra et al. 2019). Therefore, Glyma.10G019000 is suggested to be an MRP4 which predominantly functions under drought stress in soybean. AT2G47800, an Arabidopsis ortholog of Glyma.10G019000, was highly expressed in stomata and involved in the regulation of stomatal aperture (Klein et al. 2004; Gong et al. 2022). Knockout mutants of AtMRP4 showed larger stomatal aperture regardless of light and wilt faster than wild-type under drought conditions (Klein et al. 2004). Recently, it was discovered that MRP4-dependent eATP release triggered by ethyl vinyl ketone (evk) mediates K^+^ Efflux, leading to stomata closure (Gong et al. 2022). Both PK1B and MRP4 are known to be involved in the regulation of H^+^-ATPase activity, a key regulator of stomatal opening (Zhao et al. 2019). Thus, the optimal regulation of H^+^-ATPase activity by the two genes would lead to an ideal stomatal aperture balancing of gas exchange and water regulation, resulting in enhanced slow wilting.

## Conclusion

In this study, we utilized the valuable genetic resource SS2-2 showing slow wilting to identify genetic loci responsible for slow wilting. A novel QTL, *qSW_Gm01*, was identified to function stably across multiple environments, along with the previously reported QTL, *qSW_Gm10*. These two QTLs interplay synergistically, wherein the effect of their combined alleles surpassed the sum of their individual additive effects in phenotypic values of wilting score and leaf moisture content. Using multi-omics data, we propose Glyma.01G098400 (*PK1B*) and Glyma.10G019000 (*MRP4*) as the putative candidate genes for *qSW_Gm01* and *qSW_Gm10*, respectively, even though their functional validation is necessary. In conclusion, our findings provide primary insights into the genetic mechanisms underlying slow wilting in soybean and promising genetic resources to enhance slow wilting in soybean breeding programs.

### Supplementary Information

Below is the link to the electronic supplementary material.

Supplementary file1 (ZIP 103 KB)

Supplementary file2 (XLSX 22 KB)

Supplementary file3 (DOCX 8878 KB)

## Data Availability

Every in-house made Python script used in this study is available at http://plantgenomics.snu.ac.kr/mediawiki-1.21.3/index.php/Slow_wilting_QTL. The resequencing data for SS2-2 are available on NCBI with SRA ID PRJNA1058168.

## References

[CR1] Abdel-Haleem H, Carter TE, Purcell LC (2012). Mapping of quantitative trait loci for canopy-wilting trait in soybean (Glycine max L. Merr). Theor Appl Genet.

[CR2] Bazzer SK, Purcell LC (2020). Identification of quantitative trait loci associated with canopy temperature in soybean. Sci Rep.

[CR3] Blomster T, Salojärvi J, Sipari N (2011). Apoplastic reactive oxygen species transiently decrease auxin signaling and cause stress-induced morphogenic response in arabidopsis. Plant Physiol.

[CR4] Bolger AM, Lohse M, Usadel B (2014). Trimmomatic: a flexible trimmer for Illumina sequence data. Bioinformatics.

[CR5] Brown AV, Conners SI, Huang W (2021). A new decade and new data at SoyBase, the USDA-ARS soybean genetics and genomics database. Nucleic Acids Res.

[CR6] Bunce JA (1984). Identifying soybean lines differing in gas exchange sensitivity to humidity. Ann Appl Biol.

[CR7] Carpentieri-Pipolo V, Pipolo AE, Abdel-Haleem H (2012). Identification of QTLs associated with limited leaf hydraulic conductance in soybean. Euphytica.

[CR8] Charlson DV, Bhatnagar S, King CA (2009). Polygenic inheritance of canopy wilting in soybean [*Glycine max* (L.) Merr.]. Theor Appl Genet.

[CR9] Charron J-BF, Ouellet F, Houde M, Sarhan F (2008). The plant Apolipoprotein D ortholog protects Arabidopsis against oxidative stress. BMC Plant Biol.

[CR10] Chen C, Chen H, Zhang Y (2020). TBtools: an integrative toolkit developed for interactive analyses of big biological data. Mol Plant.

[CR11] Chun HC, Lee S, Choi YD (2021). Effects of drought stress on root morphology and spatial distribution of soybean and adzuki bean. J Integr Agric.

[CR12] Chung U, Seo MC, Jung WS, Kim JH, Cho HS (2015). Exploring of characteristics of relative yield change of soybean under drought events. Proc Korean Soc Agric For Meteorol Conf.

[CR13] Cingolani P, Platts A, Wang LL (2012). A program for annotating and predicting the effects of single nucleotide polymorphisms, SnpEff. Fly (austin).

[CR14] Cominelli E, Galbiati M, Vavasseur A (2005). A guard-cell-specific MYB transcription factor regulates stomatal movements and plant drought tolerance. Curr Biol.

[CR15] Danecek P, Auton A, Abecasis G (2011). The variant call format and VCFtools. Bioinformatics.

[CR16] Davletova S, Schlauch K, Coutu J, Mittler R (2005). The zinc-finger protein Zat12 plays a central role in reactive oxygen and abiotic stress signaling in Arabidopsis. Plant Physiol.

[CR17] De Block M, Verduyn C, De Brouwer D, Cornelissen M (2005). Poly(ADP-ribose) polymerase in plants affects energy homeostasis, cell death and stress tolerance. Plant J.

[CR18] Denver JB, Ullah H (2019). miR393s regulate salt stress response pathway in Arabidopsis thaliana through scaffold protein RACK1A mediated ABA signaling pathways. Plant Signal Behav.

[CR19] Devi JM, Sinclair TR, Chen P, Carter TE (2014). Evaluation of elite southern maturity soybean breeding lines for drought-tolerant traits. Agron J.

[CR20] Ding Y, Liu N, Virlouvet L (2013). Four distinct types of dehydration stress memory genes in Arabidopsis thaliana. BMC Plant Biol.

[CR21] Ea BRAY (2002). Classification of genes differentially expressed during water-deficit stress in arabidopsis thaliana: an analysis using microarray and differential expression data. Ann Bot.

[CR22] Elhaddad NS, Hunt L, Sloan J, Gray JE (2014). Light-induced stomatal opening is affected by the guard cell protein kinase APK1b. PLoS ONE.

[CR23] Fang Q, Wang Q, Mao H (2018). AtDIV2, an R-R-type MYB transcription factor of Arabidopsis, negatively regulates salt stress by modulating ABA signaling. Plant Cell Rep.

[CR24] Fletcher AL, Sinclair TR, Allen LH (2007). Transpiration responses to vapor pressure deficit in well watered ‘slow-wilting’ and commercial soybean. Environ Exp Bot.

[CR25] Gonda I, Ashrafi H, Lyon DA (2019). Sequencing-based bin map construction of a tomato mapping population, facilitating high-resolution quantitative trait loci detection. Plant Genome.

[CR26] Gong J, Yao L, Jiao C (2022). Ethyl vinyl ketone activates K+ efflux to regulate stomatal closure by MRP4-dependent eATP accumulation working upstream of H2O2 Burst in Arabidopsis. Int J Mol Sci.

[CR27] Hachez C, Ohashi-Ito K, Dong J, Bergmann DC (2011). Differentiation of arabidopsis guard cells: analysis of the networks incorporating the basic helix-loop-helix transcription factor. PFMPA Plant Physiol.

[CR28] Huang D, Wu W, Abrams SR, Cutler AJ (2008). The relationship of drought-related gene expression in Arabidopsis thaliana to hormonal and environmental factors. J Exp Bot.

[CR29] Huang K-C, Lin W-C, Cheng W-H (2018). Salt hypersensitive mutant 9, a nucleolar APUM23 protein, is essential for salt sensitivity in association with the ABA signaling pathway in Arabidopsis. BMC Plant Biol.

[CR30] Hwang S, King A, Davies M (2015). Registration of the KS4895 × Jackson Soybean mapping population, AR93705. J Plant Regist.

[CR31] Hwang S, King CA, Ray JD (2015). Confirmation of delayed canopy wilting QTLs from multiple soybean mapping populations. Theor Appl Genet.

[CR32] Hwang S, King CA, Chen P (2016). Meta-analysis to refine map position and reduce confidence intervals for delayed-canopy-wilting QTLs in soybean. Mol Breed.

[CR33] Jumrani K, Bhatia VS (2018). Impact of combined stress of high temperature and water deficit on growth and seed yield of soybean. Physiol Mol Biol Plants.

[CR34] Jung C, Seo JS, Han SW (2008). Overexpression of AtMYB44 enhances stomatal closure to confer abiotic stress tolerance in transgenic Arabidopsis. Plant Physiol.

[CR35] Kaler AS, Ray JD, Schapaugh WT (2017). Genome-wide association mapping of canopy wilting in diverse soybean genotypes. Theor Appl Genet.

[CR36] Kim SJ, Ryu MY, Kim WT (2012). Suppression of Arabidopsis RING-DUF1117 E3 ubiquitin ligases, AtRDUF1 and AtRDUF2, reduces tolerance to ABA-mediated drought stress. Biochem Biophys Res Commun.

[CR37] Kim J, Boo K-O, Choi J, Byun Y-H (2018). Climate change in the Korean peninsula over the last 100 years.

[CR38] Kim SD, Hong EH, Lee YH, Moon YH, Kim HS, Seong YG, Kim WH (1992) Resistant to disease, good in seed quality, high yielding and widely adapted new soybean variety "Taekwangkong". Research Reports of the Rural Development Administration (Korea Republic).

[CR39] Klein M, Geisler M, Suh SJ (2004). Disruption of AtMRP4, a guard cell plasma membrane ABCC-type ABC transporter, leads to deregulation of stomatal opening and increased drought susceptibility. Plant J.

[CR40] Ko J-H, Yang SH, Han K-H (2006). Upregulation of an Arabidopsis RING-H2 gene, XERICO, confers drought tolerance through increased abscisic acid biosynthesis. Plant J.

[CR41] Lee H-S, Lee S-H (1998). Introduction, development, and characterization of supernodulating soybean mutant. Korean J Crop Sci.

[CR42] Lee E, Yang X, Ha J (2021). Identification of a locus controlling compound raceme inflorescence in mungbean [Vigna radiata (L.) R. Wilczek]. Front Gen.

[CR43] Leng G, Hall J (2019). Crop yield sensitivity of global major agricultural countries to droughts and the projected changes in the future. Sci Total Environ.

[CR44] Less H, Galili G (2008). Principal transcriptional programs regulating plant amino acid metabolism in response to abiotic stresses. Plant Physiol.

[CR45] Li X, Troy TJ (2018). Changes in rainfed and irrigated crop yield response to climate in the western US. Environ Res Lett.

[CR46] Li H, Handsaker B, Wysoker A (2009). The Sequence Alignment/Map format and SAMtools. Bioinformatics.

[CR47] Li S, Wang W, Cao Y (2019). How root traits would be affected by soybean yield improvement? An examination of historical cultivars grafted with record-yield cultivar scion. Plant Soil.

[CR48] Li H (2013) Aligning sequence reads, clone sequences and assembly contigs with BWA-MEM

[CR49] Luan X, Bommarco R, Scaini A, Vico G (2021). Combined heat and drought suppress rainfed maize and soybean yields and modify irrigation benefits in the USA. Environ Res Lett.

[CR50] Luhua S, Hegie A, Suzuki N (2013). Linking genes of unknown function with abiotic stress responses by high-throughput phenotype screening. Physiol Plant.

[CR51] Meng L, Li H, Zhang L, Wang J (2015). QTL IciMapping: Integrated software for genetic linkage map construction and quantitative trait locus mapping in biparental populations. Crop J.

[CR52] Mishra AK, Choi J, Rabbee MF, Baek K-H (2019). In silico genome-wide analysis of the ATP-binding cassette transporter gene family in Soybean (Glycine max L.) and their expression profiling. Biomed Res Int.

[CR53] Money D, Migicovsky Z, Gardner K, Myles S (2017). LinkImputeR: user-guided genotype calling and imputation for non-model organisms. BMC Genom.

[CR54] Mwenye OJ, Rensburg LV, Merwe AVB and RV der, et al (2018) Seedling shoot and root growth responses among soybean (glycine max) genotypes to drought stress. In: Soybean-Biomass, yield and productivity. IntechOpen

[CR55] Pathan SM, Lee JD, Sleper DA, Fritschi FB, Sharp RE, Carter TE, Shannon JG (2014). Two soybean plant introductions display slow leaf wilting and reduced yield loss under drought. J Agron Crop Sci.

[CR56] Raghavan C, Ong EK, Dalling MJ, Stevenson TW (2006). Regulation of genes associated with auxin, ethylene and ABA pathways by 2,4-dichlorophenoxyacetic acid in Arabidopsis. Funct Integr Genomics.

[CR57] Renault H, Amrani EL, Berger A (2012). GABA transaminase deficiency impairs central carbon metabolism and leads to cell wall defects during salt stress in arabidopsis roots. Plant, Cell Environ.

[CR58] Rizhsky L, Liang H, Shuman J (2004). When defense pathways collide. The response of Arabidopsis to a combination of drought and heat stress. Plant Physiol.

[CR59] Sadok W, Sinclair TR (2010). Genetic variability of transpiration response of Soybean [*Glycine max* (L.) Merr.] shoots to leaf hydraulic conductance inhibitor AgNO_3_. Crop Sci.

[CR60] Sadok W, Sinclair TR (2010). Transpiration response of ‘slow-wilting’ and commercial soybean (*Glycine max* (L.) Merr.) genotypes to three aquaporin inhibitors. J Exp Bot.

[CR61] Sánchez J-P, Duque P, Chua N-H (2004). ABA activates ADPR cyclase and cADPR induces a subset of ABA-responsive genes in Arabidopsis. Plant J.

[CR62] Santini M, Noce S, Antonelli M, Caporaso L (2022). Complex drought patterns robustly explain global yield loss for major crops. Sci Rep.

[CR63] Sarkar S, Shekoofa A, McClure A, Gillman JD (2022). Phenotyping and quantitative trait locus analysis for the limited transpiration trait in an upper-mid south soybean recombinant inbred line population (“Jackson” × “KS4895”): high throughput aquaporin inhibitor screening. Front Plant Sci.

[CR64] Schultz J, Copley RR, Doerks T (2000). SMART: a web-based tool for the study of genetically mobile domains. Nucleic Acids Res.

[CR65] Shi H, Ye T, Han N (2015). Hydrogen sulfide regulates abiotic stress tolerance and biotic stress resistance in Arabidopsis. J Integr Plant Biol.

[CR66] Shin SO, Han WY, Lee BW (2015). Major factors for affecting to soybean yield decline in South Korea. J Korean Soc Int Agric.

[CR67] Sinclair TR, Zwieniecki MA, Holbrook NM (2008). Low leaf hydraulic conductance associated with drought tolerance in soybean. Physiol Plant.

[CR68] Sinclair TR, Messina CD, Beatty A, Samples M (2010). Assessment across the United States of the Benefits of Altered Soybean Drought Traits. Agron J.

[CR69] Sinclair TR, Devi J, Shekoofa A (2017). Limited-transpiration response to high vapor pressure deficit in crop species. Plant Sci.

[CR70] Song Q, Jenkins J, Jia G (2016). Construction of high resolution genetic linkage maps to improve the soybean genome sequence assembly Glyma1.01. BMC Genom.

[CR71] Specht JE, Hume DJ, Kumudini SV (1999). Soybean yield potential—a genetic and physiological perspective. Crop Sci.

[CR72] Strizhov N, Abrahám E, Okrész L (1997). Differential expression of two P5CS genes controlling proline accumulation during salt-stress requires ABA and is regulated by ABA1, ABI1 and AXR2 in Arabidopsis. Plant J.

[CR73] Sun S, Kim MY, Van K (2013). QTLs for resistance to Phomopsis seed decay are associated with days to maturity in soybean (Glycine max). Theor Appl Genet.

[CR74] Székely G, Abrahám E, Cséplo A (2008). Duplicated P5CS genes of Arabidopsis play distinct roles in stress regulation and developmental control of proline biosynthesis. Plant J.

[CR75] Tahir MS, Karagiannis J, Tian L (2022). HD2A and HD2C co-regulate drought stress response by modulating stomatal closure and root growth in Arabidopsis. Front Plant Sci.

[CR76] Tamura K, Peterson D, Peterson N (2011). MEGA5: molecular evolutionary genetics analysis using maximum likelihood, evolutionary distance, and maximum parsimony methods. Mol Biol Evol.

[CR77] Team R (2006). A language and environment for statistical computing. Computing.

[CR78] Thompson JD, Higgins DG, Gibson TJ (1994). CLUSTAL W: improving the sensitivity of progressive multiple sequence alignment through sequence weighting, position-specific gap penalties and weight matrix choice. Nucleic Acids Res.

[CR79] Vanderauwera S, De Block M, Van de Steene N (2007). Silencing of poly(ADP-ribose) polymerase in plants alters abiotic stress signal transduction. Proc Natl Acad Sci.

[CR80] Walley JW, Coughlan S, Hudson ME (2007). Mechanical stress induces biotic and abiotic stress responses via a novel cis-element. PLoS Genet.

[CR81] Wang Y, Tang H, DeBarry JD (2012). MCScanX: a toolkit for detection and evolutionary analysis of gene synteny and collinearity. Nucleic Acids Res.

[CR82] Weaver LM, Gan S, Quirino B, Amasino RM (1998). A comparison of the expression patterns of several senescence-associated genes in response to stress and hormone treatment. Plant Mol Biol.

[CR83] Weimer AK, Matos JL, Sharma N (2018). Lineage- and stage-specific expressed CYCD7;1 coordinates the single symmetric division that creates stomatal guard cells. Development.

[CR84] Wickham H, Chang W, Henry L, et al (2023) ggplot2: Create elegant data visualisations using the grammar of graphics

[CR85] Wu J (2019) minque: Various linear mixed model analyses

[CR86] Yang X, Kwon H, Kim MY, Lee S-H (2023). RNA-seq profiling in leaf tissues of two soybean (*Glycine max* [L.] Merr) cultivars that show contrasting responses to drought stress during early developmental stages. Mol Breed.

[CR87] Ye H, Song L, Schapaugh WT (2020). The importance of slow canopy wilting in drought tolerance in soybean. J Exp Bot.

[CR88] Yoon MY, Kim MY, Ha J (2019). QTL Analysis of Resistance to High-Intensity UV-B Irradiation in Soybean (Glycine max [L.] Merr). Int J Mol Sci.

[CR89] Zhang L, Zhou T (2015). Drought over east Asia: a review. J Clim.

[CR90] Zhao W, Jung S, Schubert S (2019). Transcription profile analysis identifies marker genes to distinguish salt shock and salt stress after stepwise acclimation in Arabidopsis thaliana and Zea mays. Plant Physiol Biochem.

[CR91] Zhu X, Troy TJ, Devineni N (2019). Stochastically modeling the projected impacts of climate change on rainfed and irrigated US crop yields. Environ Res Lett.

