# Supplementary material for: Unveiling synergistic QTLs associated with slow wilting in soybean (Glycine max [L.] Merr.)
Source: Theor Appl Genet. 2024 Mar 19;137(4):85. doi: 10.1007/s00122-024-04585-1 (PMC10951030; doi:10.1007/s00122-024-04585-1)
Supplement: Supplementary file 3 — Supplementary file3 (DOCX 8878 KB) [file 122_2024_4585_MOESM3_ESM.docx]

**Supplementary Information**

Unveiling synergistic QTLs associated with slow wilting in soybean (*Glycine max* [L.] Merr.)

Theoretical and Applied Genetics

Hakyung Kwon^1^, Moon Young Kim^1,2^, Suk-Ha Lee^1,2^*

^1^ Department of Agriculture, Forestry and Bioresources and Research Institute of Agriculture and Life Sciences, Seoul National University, Seoul, Republic of Korea

^2^ Plant Genomics and Breeding Institute, Seoul National University, Seoul, Republic of Korea

*Corresponding author. E-mail : [sukhalee@snu.ac.kr](mailto:sukhalee@snu.ac.kr);

**Supplementary Table 1** Correlation coefficients for wilting score and leaf moisture content for each year. Pearson correlation coefficient is shown with *,** and *** for *P* <0.05, 0.01 and 0.001, respectively.

|  |  | | Wilting score | | |  | Leaf moisture content | | |
| --- | --- | --- | --- | --- | --- | --- | --- | --- | --- |
|  |  | | 2019 | 2021 | 2022 |  | 2019 | 2021 | 2022 |
| Wilting score | 2019 | 1 |  |  |  |  |  |  |  |
|  | 2021 | 0.18* | 1 |  |  |  |  |  |  |
|  | 2022 | 0.31** | 0.27*** | 1 |  |  |  |  |  |
|  |  |  |  |  |  |  |  |  |  |
| Leaf moisture content | 2019 | -0.50*** | -0.20* | -0.21* |  | 1 |  |  |  |
|  | 2021 | -0.25** | -0.74*** | -0.25*** |  | 0.17* | 1 |  |  |
|  | 2022 | -0.17* | -0.22** | -0.76*** |  | 0.095 | 0.29*** | 1 |  |

**Supplementary Table 2** Summary statistics of Taekwangkong and SS2-2 resequencing data

|  | Taekwangkong | SS2-2 |
| --- | --- | --- |
| Total number of bases (Gb) | 16.0 | 17.0 |
| Number of raw reads | 106,328,788 | 168,675,062 |
| Number of bases per reads | 151 | 101 |
| Number of reads after trimming | 103,768,234 | 168,458,411 |
| Number of mapped reads | 87,603,858 | 134,538,506 |
| Average depth | 14.6626 | 13.5181 |
| Coverage | 91.21% | 92.28% |

Supplementary Table 3 Statistics of SNP marker usage

| SNP marker usage | Number of variants |
| --- | --- |
| Total number of variants | 3,491,200 |
| Total number of SNPs from population genotyping | 3,100,364 |
| Number of filtered SNPs | 21,033 |
| Number of SNPs differentiating the parental lines | 11,530 |
| Number of SNPs after preprocessing and imputation | 10,982 |
| Number of SNPs after binning | 1,403 |
| Number of bins | 1,383 |

**Supplementary Table 4** Summary statistics of the genetic bin map in the RIL population of Taekwangkong x SS2-2

| Linkage group | Number of markers | Length (cM) | Avg. marker distance (cM) |
| --- | --- | --- | --- |
| 1 | 54 | 83.82 | 1.58 |
| 2 | 84 | 107.87 | 1.30 |
| 3 | 80 | 98.35 | 1.24 |
| 4 | 72 | 116.31 | 1.64 |
| 5 | 77 | 109.36 | 1.44 |
| 6 | 89 | 116.39 | 1.32 |
| 7 | 52 | 109.88 | 2.15 |
| 8 | 77 | 108.93 | 1.43 |
| 9 | 76 | 135.48 | 1.81 |
| 10 | 57 | 100.80 | 1.80 |
| 11 | 45 | 83.47 | 1.90 |
| 12 | 58 | 84.73 | 1.49 |
| 13 | 61 | 129.84 | 2.16 |
| 14 | 51 | 71.43 | 1.43 |
| 15 | 65 | 101.32 | 1.58 |
| 16 | 77 | 83.86 | 1.10 |
| 17 | 60 | 95.92 | 1.63 |
| 18 | 91 | 97.35 | 1.08 |
| 19 | 89 | 98.40 | 1.12 |
| 20 | 68 | 109.78 | 1.64 |
| Total | 1,383 | 2,043.30 | 1.48 |

**Supplementary table 5** QTL analysis results of BIP module for slow-wilting traits using yearly averaged phenotype values in the RIL population derived from Taekwangkong x SS2-2

| Trait | year | Locus | Chr | Position (cM) | Flanking markers | LOD | PVE | Add. | Source of beneficial allele |
| --- | --- | --- | --- | --- | --- | --- | --- | --- | --- |
| Wilting score | 2019 | *qSW_Gm03* | 3 | 33 | Bin_3_33457443, Bin_3_34104446 | 2.91 | 0.78 | 0.12 | Taekwangkong |
|  |  | *qSW_Gm04_1* | 4 | 37 | Bin_4_7118893,  Bin_4_7259025 | 28.11 | 9.71 | 0.40 | SS2-2 |
|  |  | *qSW_Gm04_2* | 4 | 40 | Bin_4_8042847,  Bin_4_8126955 | 19.10 | 5.91 | -0.31 | Taekwangkong |
|  | 2021 | *qSW_Gm01* | 1 | 75 | Bin_1_52074632, Bin_1_52737077 | 4.27 | 5.96 | 0.25 | SS2-2 |
|  |  | *qSW_Gm05* | 5 | 55 | Bin_5_31865874, Bin_5_32451279 | 3.57 | 4.68 | 0.22 | SS2-2 |
|  |  | *qSW_Gm06_1* | 6 | 90 | Bin_6_48390180, Bin_6_48545387 | 3.14 | 4.23 | -0.21 | Taekwangkong |
|  |  | *qSW_Gm10* | 10 | 6 | Bin_10_1686946, Bin_10_2097501 | 9.00 | 12.51 | 0.39 | SS2-2 |
|  | 2022 | *qSW_Gm10* | 10 | 6 | Bin_10_1686946, Bin_10_2097501 | 4.50 | 12.93 | 0.21 | SS2-2 |
| Moisture content | 2021 | *qSW_Gm01* | 1 | 75 | Bin_1_52074632, Bin_1_52737077 | 4.41 | 5.55 | -0.05 | SS2-2 |
|  |  | *qSW_Gm06_2* | 6 | 96 | Bin_6_48851328, Bin_6_48920561 | 4.23 | 5.30 | 0.05 | Taekwangkong |
|  |  | *qSW_Gm09* | 9 | 52 | Bin_9_20688019, Bin_9_26672380 | 4.22 | 5.12 | -0.05 | SS2-2 |
|  | 2022 | *qSW_Gm07* | 7 | 82 | Bin_7_36046154, Bin_7_36200793 | 2.60 | 4.72 | 0.02 | Taekwangkong |
|  |  | *qSW_Gm10* | 10 | 6 | Bin_10_1686946, Bin_10_2097501 | 7.64 | 9.65 | -0.07 | SS2-2 |

**Supplementary Table 7** DEGs in *qSW_Gm01* and *qSW_Gm10* between Taekwangkong and SS2-2 under drought condition

| locus | gene ID | annotation | logFC (SS2-2/Taekwangkong) | | logCPM | *P* value | | |
| --- | --- | --- | --- | --- | --- | --- | --- | --- |
| *qSW_Gm01* | Glyma.01G098400 | protein kinase 1B | 1.63 | 2.37 | | | < 0.001 |  |
|  | Glyma.01G098700 | Haloacid dehalogenase-like hydrolase (HAD) superfamily protein | -1.98 | 6.50 | | | 0.001 |  |
|  | Glyma.01G099800 | delta 1-pyrroline-5-carboxylate synthase 2 | -5.64 | 6.08 | | | < 0.001 |  |
|  | Glyma.01G100200 | glucose-induced degradation-like protein | -1.04 | 3.07 | | | 0.003 |  |
|  | Glyma.01G104100 | isochorismate synthase 2 | 1.50 | 3.00 | | | 0.012 |  |
| *qSW_Gm10* | Glyma.10G018300 | Mitochondrial import inner membrane translocase subunit Tim17/Tim22/Tim23 family protein | -1.40 | 2.82 | | | < 0.001 |  |
|  | Glyma.10G018700 | Adenine nucleotide alpha hydrolases-like superfamily protein | -2.15 | 4.73 | | | < 0.001 |  |
|  | Glyma.10G021300 | Thioredoxin superfamily protein | 2.45 | 3.64 | | | 0.019 |  |
|  | Glyma.10G021700 | arabinogalactan protein 26 | 3.11 | 2.45 | | | 0.002 |  |
|  | Glyma.10G022900 | alpha/beta-Hydrolases superfamily protein | -1.10 | 4.60 | | | 0.005 |  |
|  | Glyma.10G023200 | histidine-containing phosphotransfer factor 5 | -1.19 | 3.03 | | | 0.001 |  |
|  | Glyma.10G024200 | hypothetical protein | 1.04 | 6.64 | | | 0.032 |  |


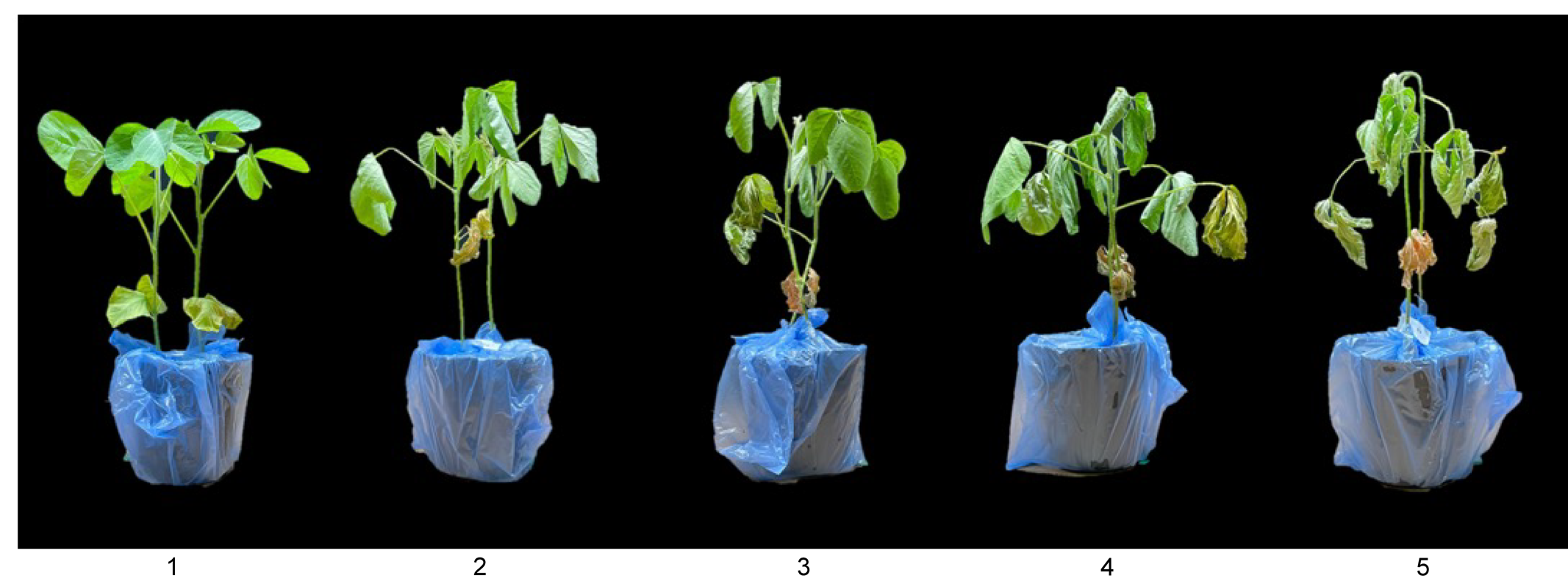
**Supplementary Fig. 1** Grading scale used for visual inspection of wilting in the RILs of Taekwangkong x SS2-2

**Supplementary Fig. 2** Distribution and correlations of the slow-wilting traits for each year. The orange color represents wilting score, while light blue represents leaf moisture content. Yellow and green arrows indicate the fast-wilting cultivar, Taekwangkong, and the slow-wilting cultivar, SS2-2, respectively. *, ** and ***, denote significance at *P* < 0.05, 0.01, and 0.001, respectively


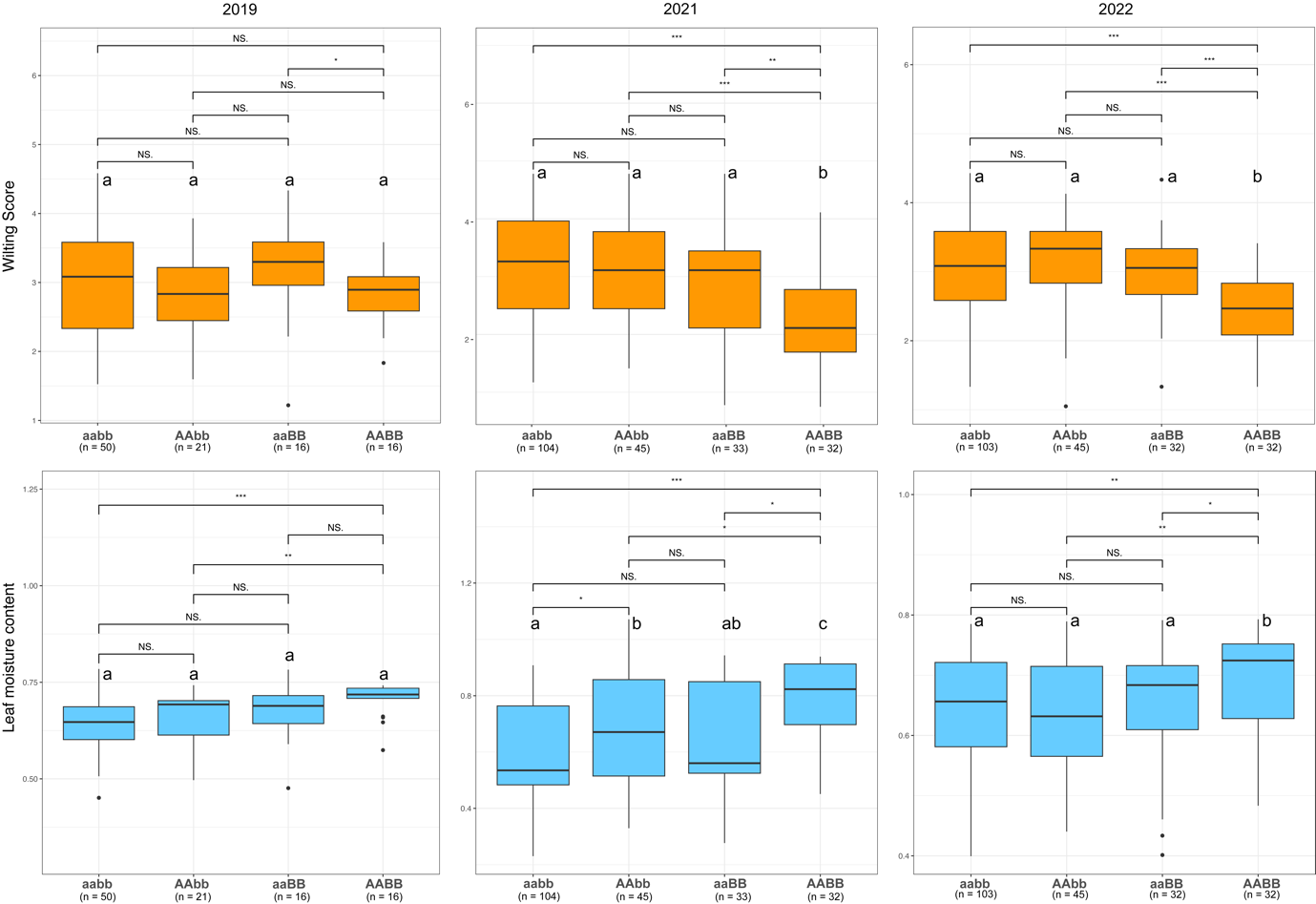


**Supplementary Fig. 3** Allelic effects of two loci, *qSW_Gm01* and *qSW_Gm10*, on wilting score and leaf moisture content in the RIL population for each year. Beneficial alleles from SS2-2 are represented by capital letters, while alleles from Taekwangkong are denoted by lowercase letters. "A" and "B" represent *qSW_Gm01* and *qSW_Gm10*, respectively. Differences between allele combinations were evaluated using the Wilcoxon test. *, ** and ***, denote significance at *P* < 0.05, 0.01, and 0.001, respectively

**Supplementary Fig. 4** Survey of candidate genes with loss-of-function variants in *qSW_Gm01* and *qSW_Gm10*. The number of genes for each filtering criterion is shown
